# Successful treatment of pediatric acute phosphate nephropathy: a case report and literature review

**DOI:** 10.3389/fmed.2026.1803319

**Published:** 2026-03-25

**Authors:** Siyi Zhu, Lei Sun, Xinyu Kuang, Ping Wang, Yulin Kang, Ying Wu, Wenyan Huang

**Affiliations:** Department of Nephrology, Rheumatology and Immunology, Shanghai Children’s Hospital, School of Medicine, Shanghai Jiao Tong University, Shanghai, China

**Keywords:** acute phosphate nephropathy, calcium and phosphorus metabolism, children, crystalline nephropathy, renal calcium deposition

## Abstract

**Background:**

Acute phosphate nephropathy is a type of acute kidney injury caused by the deposition of calcium phosphate crystals in the renal tubules- and interstitium. Currently, there have been no reported cases of acute phosphate nephropathy in children. Its typical manifestations include frequent urination, urgency, foamy urine, and impaired renal function, which is often accompanied by hypercalcemia and hypercalciuria, while patients may also experience severe symptoms such as acute renal failure.

**Case report:**

This article reports a case of acute phosphate nephropathy in a 9-year and 4-month-old child most likely caused by long-term use of alpha-calcidol. The child presented with frequent urination, urgency, foamy urine, elevated blood calcium and urinary calcium, increased blood creatinine, and urinary ultrasound suggested possible calcium salt deposition in the renal pyramids of both kidneys. Renal biopsy indicated a significant deposition of calcium phosphate salts in the renal tubular interstitium. After combined traditional Chinese and Western medicine treatment, the child’s blood calcium, urinary calcium, and blood creatinine levels gradually decreased to normal.

**Conclusion:**

This first reported case of acute phosphate nephropathy in child was possibly caused by long-term use of alpha-calcidol. The patient achieved good results following a comprehensive treatment plan combining traditional Chinese and Western medicine. This approach not only promoted the recovery of renal function but also offered valuable insights for clinical practice. Additionally, recent literature is reviewed to discuss the etiology, pathogenic mechanisms, and treatment strategies of this disease, aiming to enhance clinical physicians’ awareness and vigilance regarding such conditions.

## 1 Introduction

Crystalline nephropathy refers to a class of diseases characterized by the massive deposition of various crystals in the renal tubules and interstitium, leading to renal dysfunction (1). Acute phosphate nephropathy is an acute kidney injury caused by the deposition of calcium phosphate crystals in the renal tubules-interstitium, which is extremely rare and cannot be expelled through drug treatment, relying solely on self-absorption. Currently reported adult cases are mostly seen after oral sodium phosphate (OSP) bowel laxative prior to colonoscopy (2), and no reports have been found in children. Although studies on adult cases have been conducted, the clinical characteristics and potential mechanisms of acute phosphate nephropathy in children have not been fully understood, making research on this disease of significant clinical value. Children have a weaker ability to regulate the excretion of calcium and phosphate in the renal tubules, and excessive supplementation of vitamin D and its analogs can further inhibit parathyroid hormone (PTH), thereby increasing the risk of hypercalcemia and hypercalciuria (3). This article reports a case of acute phosphate nephropathy in a child probably caused by long-term use of α-calcidol, which was successfully diagnosed and treated, while also discussing its etiology, pathogenesis, and treatment strategies based on recent literature, which helps improve clinical physicians’ understanding of this disease and provides important references for future diagnosis and management.

## 2 Case presentation

### 2.1 Patient information

This case involves a 9-year-and-4-month-old male patient, who was admitted due to “frequent urination accompanied by foamy urine for more than half a year, and elevated blood creatinine (Serum creatinine) for 1 day.” The current medical history shows that the patient experienced frequent urination and urgency in October 2023 without obvious inducement, accompanied by increased nocturia and foamy urine, along with polydipsia, without dysuria or gross hematuria, and no special treatment was given. On April 16, 2024, an external hospital’s blood gas analysis showed a pH of 7.538, calcium 1.69 mmol/L, and no abnormalities in routine urine tests; urine analysis: calcium 3.84 mmol/L, CA/CR ratio 0.442, and ultrasound of the urinary system suggested possible calcium salt deposition in both renal pyramids. Due to an unclear diagnosis, the patient was transferred to our hospital for further diagnosis and treatment on April 19, 2024, with an outpatient blood creatinine level of 126 μmol/L, diagnosed with “hypercalciuria, acute kidney injury” and admitted. No significant gastrointestinal symptoms were observed, such as nausea and vomiting or loss of appetite, nor were there neurological symptoms like fatigue, drowsiness, or confusion, or other musculoskeletal or cardiovascular symptoms. The patient’s past medical history includes oral administration of α-calcidol 2-3 μg/day since February 2023 for 1 year; in March 2023, an external hospital’s blood biochemistry showed creatinine 50 μmol/L. In 2023, whole exome sequencing (WES) did not indicate relevant mutations. In terms of family history, the patient’s grandmother has a history of kidney stones, and other tests showed that the home water filter quality is normal, at 0.04 ppm.

### 2.2 Clinical findings

#### 2.2.1 Laboratory tests

1. Liver and kidney function, electrolytes: In blood biochemistry: creatinine 114 μmol/L (46–63), calcium 2.88 mmol/L (2.2–2.75), phosphorus 0.90 mmol/L (0.84–1.85), AP 207 U/L (86–315), liver function normal, other electrolytes normal; blood gas indicates mild metabolic alkalosis.
2. Bone metabolism, endocrine: PTH 0.87 pmol/L (1.58–6.83), osteocalcin 85.85 ng/mL (14–46), β-collagen degradation products 0.82 ng/mL (0.04–0.78), 25-OH-vitD 47.63 nmol/L (<37.5, deficiency; 37.5–74.9, insufficiency; 75–250, adequacy; >250, excess); thyroid function is normal.
3. Osmotic pressure: blood osmotic pressure: 277 mOsm/kg; urine osmotic pressure: 333 mOsm/kg.
4. Urine analysis: routine urine: urine protein negative, urine red blood cells 0/HP, urine Ca/Cr fluctuating between 0.29–0.52 (0–0.21), urine series protein shows elevated tubular protein, 24-h urine biochemistry: urine protein 0.13–0.14 g (0–0.15), urine calcium 4.35–6.34 mmol/24 h.
5. Metabolism: blood and urine tandem mass spectrometry, ceruloplasmin are normal.
6. Viral infection: EB, TORCH, hepatitis B and C viruses are all normal.
7. Immune-related: humoral immunity, complement, cellular immunity, and autoantibodies are all normal.

##### 2.2. 2 Special examinations

**Figure 1:**
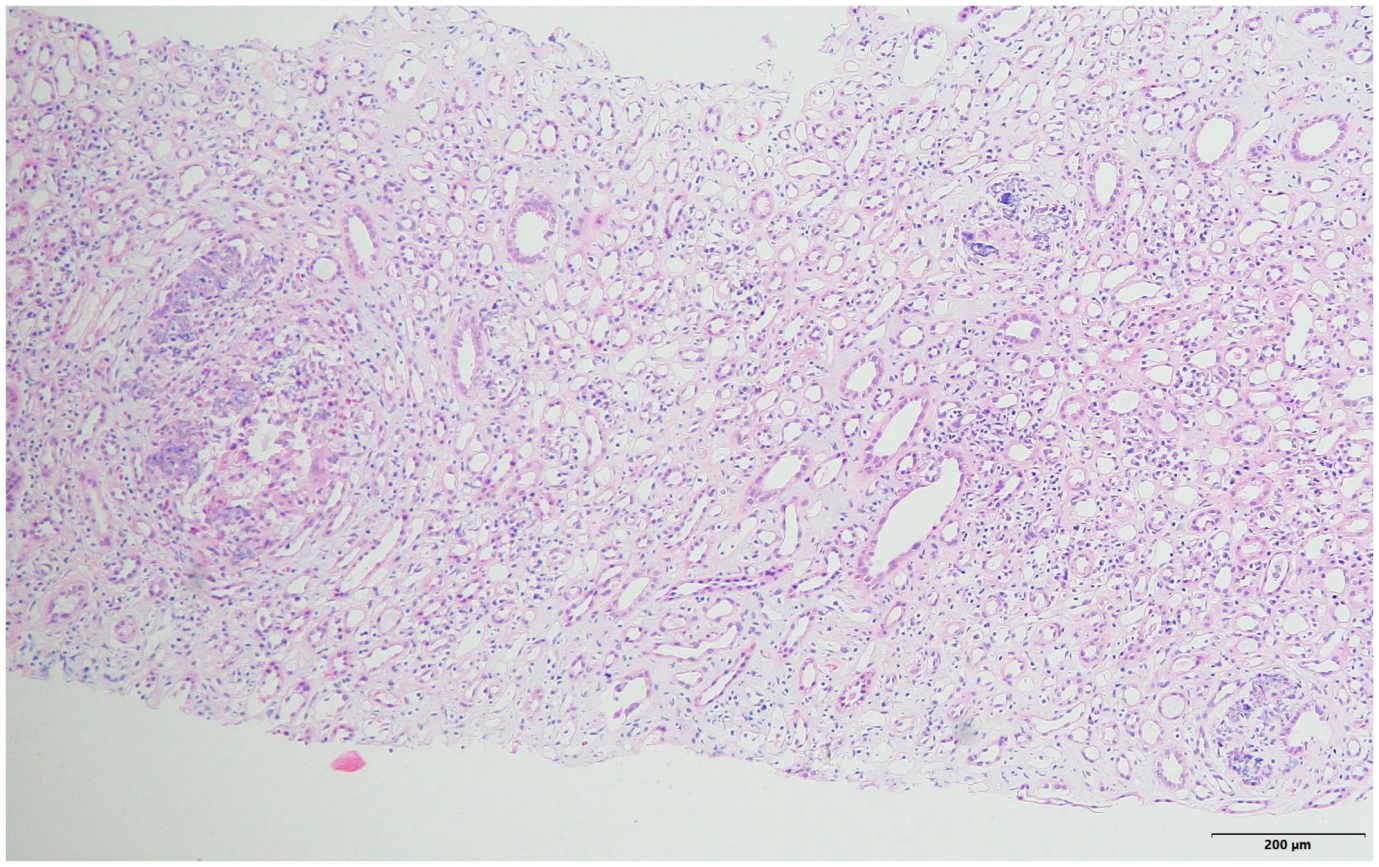
HE staining: mild renal tubule interstitial lesions, a small number of tubule epithelial cells flattened, brush border shedding, a small number of tubule base membrane thickening, atrophic half of the surrounding interstitial fibrosis, tubule lumen and interstitial area can be seen more light blue basophilic particles under HE staining (×100).

**Figure 2:**
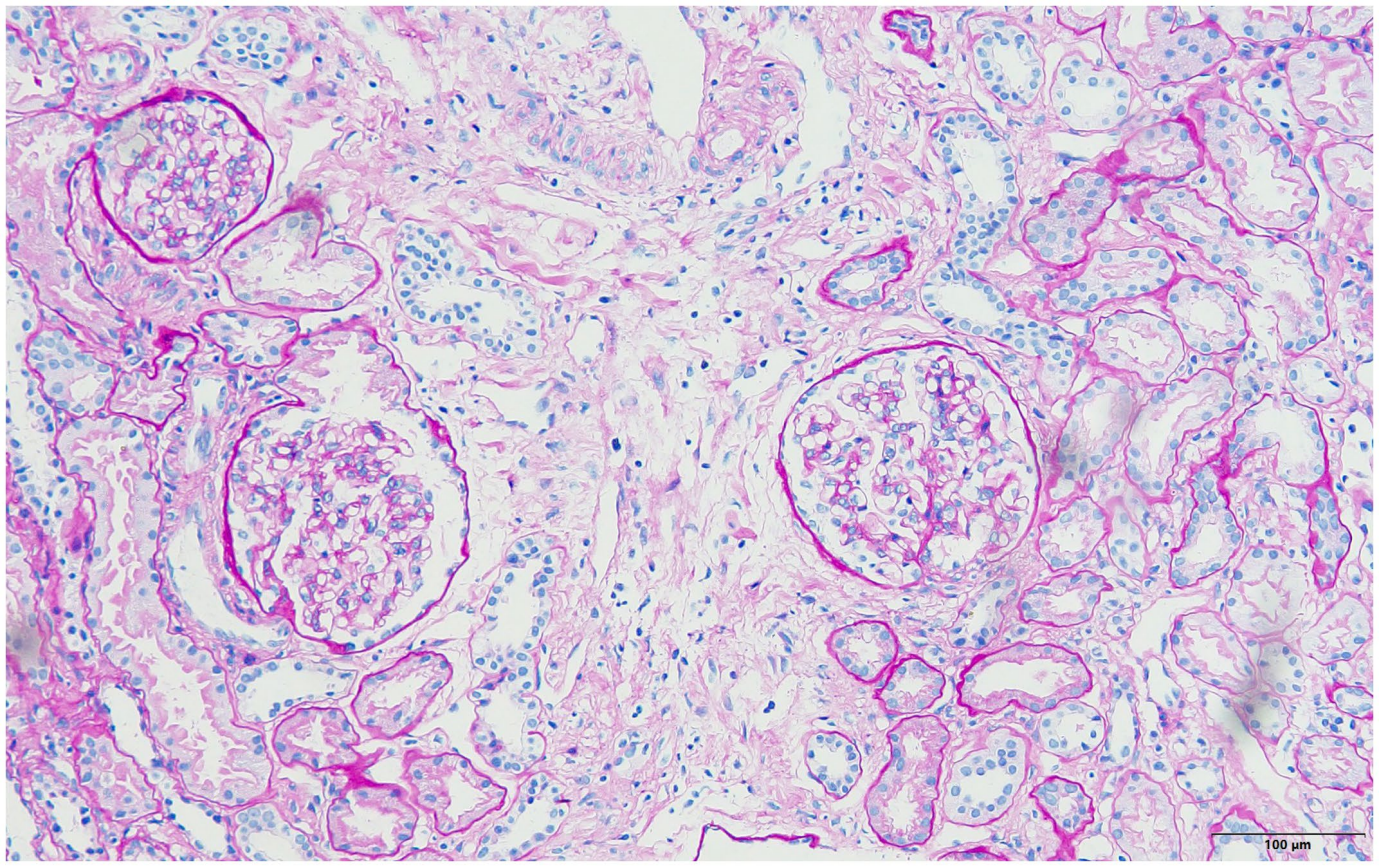
PAS staining: showed that the glomerular capillary loop was well opened, the mesangial region was not widened, the mesangial cells and endothelial cells were not proliferated, the basal membrane was not thickened, balloon adhesion and segmental sclerosis were not observed, and the wall of part of the glomerular balloon was thickened (×200).

**Figure 3:**
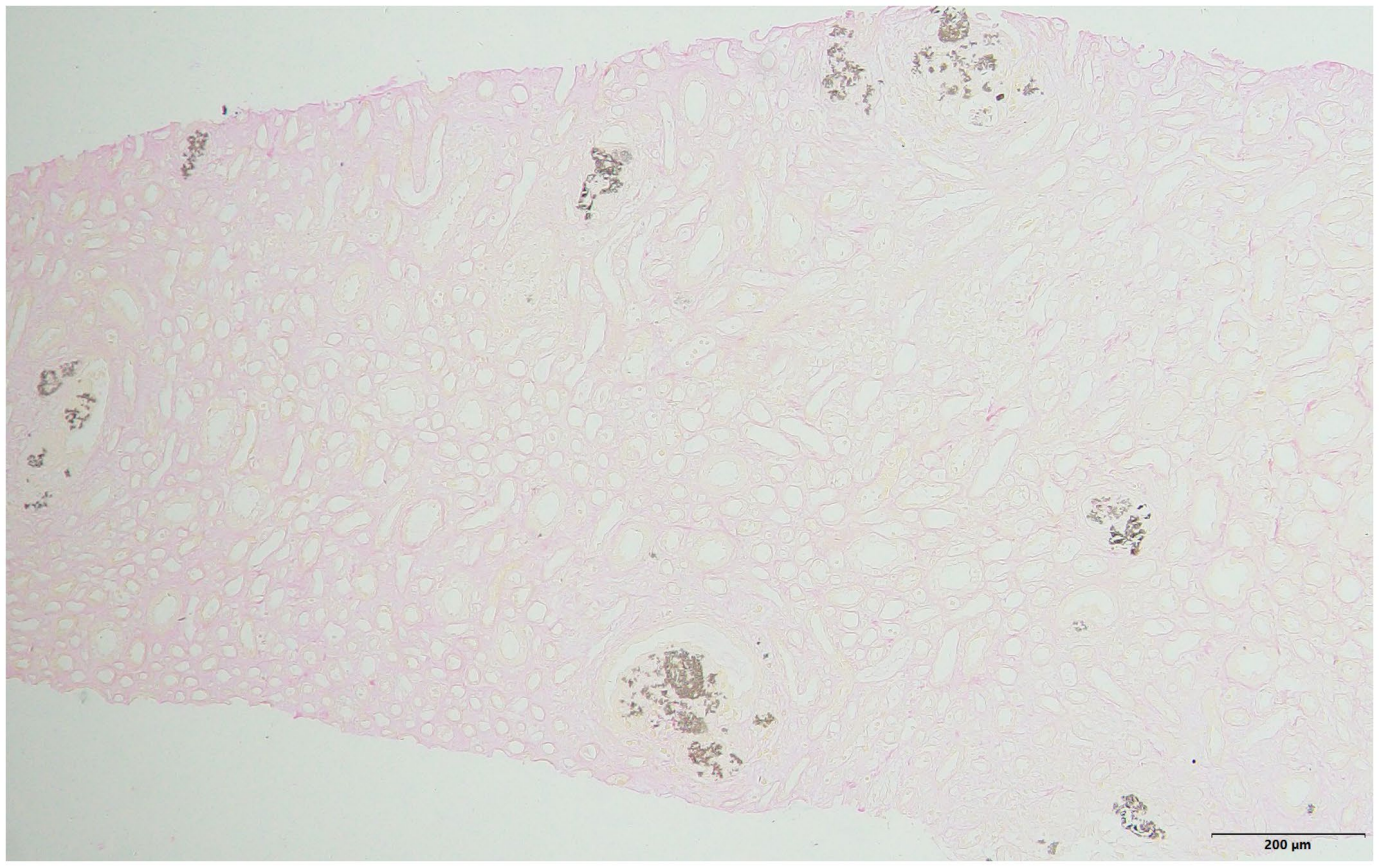
Von Kossa staining: more Von Kossa staining + particle deposits can be seen in the lumen and interstitial area of renal tubules, and some large samples are aggregated into clusters and distributed in clumps (×100).

1. Ophthalmology and hearing tests are normal.
2. Nuclear medicine examination: renal dynamic and static tests are normal.
3. Imaging examination: urinary system B ultrasound suggests possible calcium salt deposition in both renal pyramids. No plain radiography or computed tomography (CT) was performed.
4. Renal puncture biopsy (April 24, 2024): pathology suggests significant calcium phosphate salt deposition in the renal tubules (see Figures 1–3), considering crystalline kidney disease.

## 3 Diagnosis, treatment, and prognosis

The patient presents with bladder irritation symptoms such as frequent urination and urgency, along with clinical manifestations like foamy urine and polydipsia. Laboratory tests indicate hypercalcemia and hypercalciuria. Imaging studies: renal ultrasound shows calcium salt deposition in the renal pyramids of both kidneys, and renal biopsy reveals significant calcium phosphate deposition in the renal tubulointerstitium.

After comprehensive evaluation, the patient was diagnosed with acute calcium phosphate nephropathy, acute kidney injury (AKIN stage I), hypercalcemia, and hypercalciuria. Acute Phosphate Nephropathy is an acute kidney injury caused by the deposition of calcium phosphate crystals in the renal tubules and interstitium, mainly related to calcium-phosphorus metabolism imbalance. The patient’s previous history of alpha-calcidol supplementation may be the main reason for this onset.

The treatment plan includes discontinuation of alpha-calcidol and a combination of traditional Chinese and Western medicine to protect renal function, promote blood circulation, and improve circulation. Western medicine treatment includes: intravenous hydration, oral administration of Shenyan Kangfu Pian and Compound α-Keto Acid Tablets to protect the kidneys and delay the deterioration of renal function. Traditional Chinese Medicine (TCM) treatment primarily involves prescribed oral herbal decoction, containing ingredients such as White Poria, Raw Rehmannia Root, Chuanxiong, Raw White Peony Root, Green Forsythia, Honey-fried Licorice, Wolfberry Fruit, and Reed Rhizome, with gradual addition of White Chrysanthemum Flower, Magnolia Flower, and Processed Schisandra Fruit, etc.; the main effects are to enhance blood circulation and remove blood stasis, as well as to replenish qi and tonify the kidneys.

**Figure 4:**
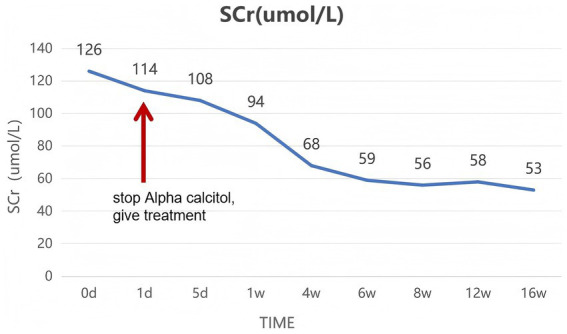
The change curve of serum creatinine (Scr, μmol/L). The patient’s serum creatinine level has significantly decreased, and acute kidney injury is in remission.

**Figure 5:**
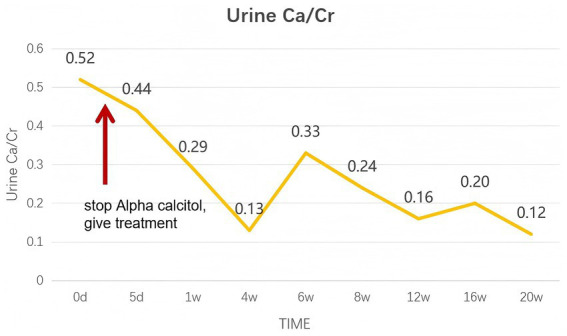
The change curve of urinary calcium/creatinine (Ca/Cr). The patient’s urine calcium/creatinine decreased, and the hypercalciuria condition was relieved.

After receiving treatment, the patient’s symptoms significantly improved within a week, and after a month of treatment, blood calcium, urinary calcium, and renal function all returned to normal. Follow-up B-ultrasonic examination showed enhanced echo in the bilateral renal medulla. The patient was followed up regularly in the outpatient clinic at 1 week, 4 weeks, 6 weeks, 8 weeks, 12 weeks, 16 weeks, and 20 weeks, during which blood creatinine and urinary calcium/creatinine indicators showed good improvement, with specific trends shown in the figure (Figures 4, 5).

## 4 Literature review and discussion

### 4.1 Discussion on etiology and pathogenesis

Currently, there have been no reports of acute calcium phosphate nephropathy in children. In adult case reports, the main trigger for acute calcium phosphate nephropathy is the use of oral sodium phosphate solution (OSPS) as a bowel cleanser before colonoscopy (4). The small intestine has a relatively weak ability to absorb and regulate phosphate, making it difficult to respond quickly to excessive phosphate intake. Meanwhile, renal tubules can adjust the amount of phosphate reabsorption within minutes, leading to a decrease in reabsorption in the proximal renal tubules, while reabsorption in the distal renal tubules increases rapidly (5), resulting in an increase and deposition of calcium-phosphate products in the renal tubular lumen, forming calcium phosphate crystals. When these calcium phosphate crystals accumulate in large quantities, they may cause tubular damage and renal dysfunction.

During a thorough inquiry into the child’s medical history, attention was paid to whether there was a possibility of phosphate intake. The assessment of the patient’s hydration status upon admission showed a normal volume status. Regarding medication factors, the child had been taking alpha-calcitriol 2-3 μg/day for over a year due to attention deficit hyperactivity disorder. In terms of dietary factors, the child’s diet did not contain phosphate components. Water quality testing showed that after replacing the filter membrane in the home water purifier, the water quality was tested at 0.04 ppm (normal range). Genetic factors: The child previously underwent whole-exome sequencing (WES) testing, which showed no abnormalities, but genetic susceptibility testing was not performed. Therefore, it is considered that the cause of the condition is related to oral α-calcitriol, while the influence of water quality factors before replacing the filter membrane cannot be completely ruled out.

Alpha-calcitriol (active vitamin D3) is the active form of vitamin D metabolism, mainly mediating its physiological functions through the vitamin D receptor (VDR). Its core role is to regulate calcium and phosphate metabolism, promoting the absorption of calcium and phosphate in the intestine, thereby maintaining the homeostasis of blood calcium and phosphate, and supporting bone mineralization. Specifically, alpha-calcitriol enhances the expression of calcium-binding proteins by binding to VDR in intestinal epithelial cells, promoting the absorption of calcium ions through intestinal epithelial cells into the bloodstream, and the absorption of phosphate is also co-regulated. In the metabolic process, the precursor of alpha-calcitriol is initially converted to 25-hydroxyvitamin D3 (25(OH)D3) in the liver by 25-hydroxylase, which is the main storage form in circulation. Subsequently, 25(OH)D3 is further hydroxylated in the kidneys by 1α-hydroxylase to produce alpha-calcitriol (1,25(OH)2D3), a process regulated by various factors such as blood calcium, blood phosphate, and parathyroid hormone (PTH) to ensure the balance of calcium and phosphate in the body. Through this complex metabolic pathway, alpha-calcitriol can flexibly regulate the body’s absorption and utilization of calcium and phosphate, maintaining bone health and the stability of mineral metabolism (6).

Existing studies indicate that the renal tubular excretion regulation ability for calcium and phosphate in children is relatively weak, and excessive vitamin D intake may further inhibit the secretion of parathyroid hormone (PTH), thereby exacerbating hypercalcemia and hypercalciuria (7). Excessive intake of alpha-calcitriol can lead to disruption of its physiological regulatory mechanisms, with the most direct manifestation being a significant enhancement of intestinal absorption of calcium and phosphate, resulting in markedly elevated levels of blood calcium and phosphate (8).

The coexistence of hypercalcemia and hyperphosphatemia increases the calcium-phosphate product, creating favorable conditions for the deposition of calcium phosphate salts. Especially in the kidneys, excessive calcium phosphate salts are prone to deposit in the renal tubules and interstitium, forming microcrystals, which can lead to renal tissue damage (9, 10). This deposition of calcium phosphate salts can not only physically obstruct the renal tubules but may also induce local cellular stress responses, triggering oxidative stress and cell death pathways, ultimately leading to acute renal function impairment (11).

The occurrence of acute calcium phosphate nephropathy is primarily due to the deposition of calcium phosphate crystals within renal tubular cells and the resulting cellular damage. The internalization of calcium phosphate microcrystals leads to a disruption of calcium ion homeostasis in renal tubular epithelial cells, triggering a sustained increase in intracellular calcium concentration, activating endoplasmic reticulum stress and oxidative stress responses (12). This series of stress responses further activates various inflammatory signaling pathways, promoting apoptosis and necrosis of renal tubular epithelial cells, while also inducing the expression of pro-fibrotic factors, driving the process of renal interstitial fibrosis (11).

### 4.2 Diagnosis and differential diagnosis

*Differential diagnosis*: there are many diseases that can cause calcium salt deposition in the kidneys. Primary diseases include renal tubular interstitial diseases such as Dent disease, Bartter syndrome, Fanconi syndrome, distal renal tubular acidosis, etc.; and calcium and phosphorus metabolism diseases such as primary hyperoxaluria and primary hyperparathyroidism; secondary diseases include vitamin D toxicity, tumor-related factors, etc. It is also necessary to differentiate from other diseases that cause acute kidney injury (AKI), including acute tubular necrosis, other types of acute interstitial nephritis, and rapidly progressive glomerulonephritis.

The child underwent whole exome sequencing (WES) and urine hematuria tandem mass spectrometry without abnormalities, and ophthalmological and audiological examinations were normal. Given the severity of acute kidney injury (AKI), the unclear etiology after initial investigations, and the need to rule out treatable glomerular diseases, a decision was ultimately made to proceed with a kidney biopsy. Ultimately, the biopsy revealed extensive calcium phosphate deposition in the renal tubulointerstitium, confirming the diagnosis of Acute Phosphate Nephropathy (APN). This effectively ruled out primary glomerulonephritis or other causes of severe interstitial nephritis.

Diagnostic criteria (13):

1. Clinical manifestations: bladder irritation: frequent urination, urgency, dysuria; renal injury: hematuria; acute renal failure: including fatigue, reduced urine output (oliguria or anuria), and edema due to renal function decline. In addition, hypercalcemia, as an important accompanying symptom of this disease, can lead to a series of systemic manifestations such as nausea, vomiting, altered consciousness, and even coma (13).
2. Laboratory tests: elevated serum calcium (hypercalcemia) and elevated phosphate levels, alkaline phosphatase levels may also be abnormal, reflecting changes in bone metabolism. Renal function indicators such as serum creatinine and blood urea nitrogen are significantly elevated, indicating renal impairment. In urine analysis, indicators related to renal tubular injury are elevated, which may manifest as increased cellular casts in urine, mild proteinuria, etc., indicating damage to renal tubular epithelial cells. In addition, reduced urine output and changes in urine specific gravity both suggest renal dysfunction (13).
3. Imaging examinations: imaging examinations play an important role in the diagnosis of acute calcium phosphate nephropathy. Renal ultrasound can show enhanced echogenicity of renal parenchyma, and calcification foci may be seen in some patients, indicating calcium salt deposition. CT scan has a higher detection rate for calcification foci and can clearly show the extent and distribution of calcification within the kidneys, assisting in diagnosis.
4. Renal biopsy is the gold standard for diagnosing acute phosphate calcium nephropathy (APCN), with its core value lying in the direct observation of characteristic phosphate calcium crystal deposits in renal tissue and the comprehensive assessment of the degree of acute kidney injury caused thereby. Pathological examination can reveal a large number of calcium phosphate crystals deposited in the renal tubules and interstitium, accompanied by interstitial inflammatory cell infiltration and damage to renal tubular epithelial cells. Special staining such as Von Kossa staining can confirm the presence of calcium phosphate (gold standard) (11). Such pathological changes directly reflect the mechanical obstruction and secondary inflammatory response caused by crystal deposition (14). Another irreplaceable value of kidney biopsy lies in its differential diagnostic capability. For patients with acute kidney injury (AKI), clinical manifestations and laboratory findings may overlap with other kidney diseases, such as acute tubular necrosis, acute interstitial nephritis, or glomerular diseases. Histopathological evidence obtained through kidney biopsy allows direct observation and exclusion of these coexisting or similar renal lesions, thereby confirming that APN is the dominant factor leading to renal function deterioration. This is crucial for formulating precise treatment plans (15). Indications for renal biopsy: (1) First, when a patient presents with typical high-risk factors (such as excessive intake of phosphorus-containing drugs or vitamin D, tumor lysis syndrome, etc.) and develops acute kidney injury, but laboratory tests show that hyperphosphatemia or hypercalcemia is not obvious or atypical, leading to clinical diagnostic uncertainty, kidney biopsy should be considered to clarify the etiology (14); (2) Second, if after discontinuing suspected causative drugs (such as phosphorus-containing laxatives or vitamin D supplements) and actively correcting metabolic disorders such as hyperphosphatemia and hypercalcemia, the patient’s renal function fails to recover as expected or continues to deteriorate, kidney biopsy is crucial for excluding other potential, treatable renal pathological changes (such as interstitial nephritis or glomerular diseases). This helps in adjusting treatment strategies (15); (3) Third, for patients with complex clinical presentations, such as simultaneous exposure to multiple nephrotoxic drugs, a background of autoimmune diseases, or a transplanted kidney status, the etiology of kidney injury may be multifactorial and intertwined. In such cases, the pathological evidence provided by kidney biopsy is key to clarifying the dominant injury factor and guiding individualized treatment (16). Finally, when non-invasive test results are ambiguous or contradictory and cannot provide sufficient confidence for clinical decision-making, kidney biopsy is the ultimate means to obtain a definitive diagnosis (17). However, for cases with a recent history of oral sodium phosphate laxatives, combined with severe hyperphosphatemia, hypocalcemia, and acute kidney injury (AKI), a clinical presumptive diagnosis can still be made. If the patient’s clinical presentation aligns with typical features and the condition improves rapidly after supportive treatment, or if there are absolute contraindications, biopsy may be considered to be omitted.

### 4.3 Treatment

#### 4.3.1 Drug adjustment

First, alpha-calcidol and other medications that may increase the calcium and phosphorus load in the body should be immediately discontinued to reduce further exacerbation of calcium and phosphorus deposition. After stopping the medication, the treatment plan should be reasonably adjusted based on blood calcium and phosphorus levels and renal function status.

#### 4.3.2 Symptomatic treatment

Supportive therapy is an important component of the treatment for acute phosphate nephropathy, with the main goal of promoting the excretion of calcium and phosphorus in the body and maintaining water and electrolyte balance, including fluid replacement support or diuresis, etc. Pharmacotherapy primarily focuses on kidney protection, including drugs such as Shenyan Kangfu Pian and Compound α-Keto Acid. Integrated traditional Chinese and Western medicine treatment promotes blood circulation and removes stasis, increases renal blood flow and urine output, facilitates the excretion and absorption of calcium and phosphorus salts, alleviates calcium salt deposition in renal tubules, and protects the kidneys (18). For patients with severely impaired renal function who develop acute renal failure, dialysis treatment is a necessary supportive measure. Dialysis can effectively remove calcium, phosphorus, and metabolic waste from the blood, maintain water and salt balance in the body, and improve the clinical symptoms of patients (19). This patient did not require dialysis therapy.

## 5 Conclusion

Acute calcium phosphate nephropathy, as a severe pathological state of the kidneys caused by calcium and phosphorus metabolism imbalance, has a complex and diverse pathogenic mechanism. Current cases show that long-term excessive use of α-calcitriol is one of the important inducements of this disease, mainly by disrupting the balance of calcium and phosphorus metabolism in the body, promoting calcium and phosphorus deposition in the kidneys, thereby causing renal tissue damage. This finding not only deepens our understanding of the pathogenic mechanisms of this disease but also provides key evidence for clinical diagnosis and treatment. However, the specific pathways and regulatory mechanisms of alpha-calcitriol in acute calcium phosphate nephropathy have not been clearly defined in existing studies. Currently, there are no reported cases in children. This article reports a case of acute calcium phosphate nephropathy in a child that was successfully diagnosed and treated, and reviews its etiology, pathogenic mechanisms, diagnosis, and treatment, aiming to deepen the understanding of this disease and provide clinical doctors with scientific and reasonable diagnostic and treatment guidance to promote early identification and standardized management of patients.

## Data Availability

The original contributions presented in the study are included in the article/supplementary material, further inquiries can be directed to the corresponding author.
